# Resetting of the Baroreflex Control of Sympathetic Vasomotor Activity during Natural Behaviors: Description and Conceptual Model of Central Mechanisms

**DOI:** 10.3389/fnins.2017.00461

**Published:** 2017-08-15

**Authors:** Roger A. L. Dampney

**Affiliations:** Physiology, School of Medical Sciences, The University of Sydney Sydney, NSW, Australia

**Keywords:** baroreflex resetting, sympathetic vasomotor activity, logistic function curves, behavioral state, central baroreflex pathways, models of baroreflex function

## Abstract

The baroreceptor reflex controls arterial pressure primarily via reflex changes in vascular resistance, rather than cardiac output. The vascular resistance in turn is dependent upon the activity of sympathetic vasomotor nerves innervating arterioles in different vascular beds. In this review, the major theme is that the baroreflex control of sympathetic vasomotor activity is not constant, but varies according to the behavioral state of the animal. In contrast to the view that was generally accepted up until the 1980s, I argue that the baroreflex control of sympathetic vasomotor activity is not inhibited or overridden during behaviors such as mental stress or exercise, but instead is reset under those conditions so that it continues to be highly effective in regulating sympathetic activity and arterial blood pressure at levels that are appropriate for the particular ongoing behavior. A major challenge is to identify the central mechanisms and neural pathways that subserve such resetting in different states. A model is proposed that is capable of simulating the different ways in which baroreflex resetting is occurred. Future studies are required to determine whether this proposed model is an accurate representation of the central mechanisms responsible for baroreflex resetting.

## Introduction

The baroreceptor reflex acts as a negative feedback control system, tending to reduce the fluctuations in arterial pressure that would otherwise occur as a consequence of external disturbances, such as changes in posture or stressful stimuli. It is very important to note, however, that arterial pressure is not regulated around a constant level, but instead typically exhibits substantial and sustained variations that are associated with changes in behavior. For example, there are diurnal variations in arterial pressure, associated with changes in activity or arousal (Veerman et al., 1995). In addition, substantial increases in arterial pressure, sympathetic activity, and heart rate occur during exercise, or during stress and arousal (Dampney et al., 2008; Dampney, 2015). As pointed out by Raven et al. (2006), it was generally believed until 1980 that the parallel increases in arterial pressure, sympathetic activity and heart rate during exercise indicated that the baroreceptor reflex was either overridden or switched off. Similarly, it was also generally believed at that time that the baroreceptor reflex was also strongly inhibited during stress or arousal, thus allowing an increase in arterial pressure, sympathetic activity and heart rate under these conditions (Hilton, 1982).

In this short review I shall argue that the baroreceptor reflex is not inhibited or overridden during behaviors such as exercise or stress, but instead is reset in such a way that the reflex continues to play a critical role in regulating the arterial pressure around a level that is appropriate for the particular behavior. I shall also discuss the central mechanisms that subserve resetting of the baroreceptor reflex in different states, and present a simple conceptual model of the resetting mechanism. First, however, I shall briefly consider some general properties of the baroreceptor reflex, and the methods that are used to measure the effectiveness of the reflex.

## General properties and measurement of the baroreceptor reflex

Studies in human subjects by Raven and co-workers have shown that under resting conditions the initial changes in arterial pressure reflexly evoked by changes in baroreceptor input are due entirely to changes in heart rate and cardiac output, but after 6–8 s the reflex change in arterial pressure is due predominantly to changes in total peripheral resistance (Raven et al., 2006). Further, in exercising humans the contribution of changes in total peripheral resistance to baroreflex evoked changes in arterial pressure during the sustained phase of the response is even greater, virtually 100% (Raven et al., 2006). It is clear, therefore, that it is the vasomotor component of the reflex, rather than the cardiac component, that is of critical importance in regulating arterial pressure, at least under conditions where there is a sustained change in state from resting conditions (e.g., during exercise, mental stress, or sleep).

Furthermore, the sensitivity of the cardiac baroreflex does not reflect the sensitivity of the reflex as a whole. In conscious humans at rest, changes in carotid sinus transmural pressure induced using a neck chamber result in reflex changes in both heart rate and arterial pressure, but there is a poor correlation between the reflex heart rate and blood pressure responses (Ludbrook et al., 1980). Furthermore, the reflex bradycardia evoked by an increase in carotid sinus transmural pressure was reduced during isometric exercise as compared to rest, whereas the reflex decrease in arterial pressure was unchanged (Ludbrook et al., 1978, 1980). Similarly, in essential hypertension the gain of the cardiac baroreflex is reduced (Bristow et al., 1969), but not the gain of the baroreflex control of muscle sympathetic activity or vascular resistance (Mancia et al., 1978; Rea and Hamdan, 1990; Grassi et al., 1998). Conversely, in patients with sleep apnea the gain of the baroreflex control of muscle sympathetic activity is reduced, while the gain of the cardiac baroreflex is unchanged (Narkiewicz et al., 1998). In summary, estimates of the gain or sensitivity of the cardiac baroreflex alone provide little information about the effectiveness of the baroreflex as a whole in regulating arterial pressure. This is an important point, because in many cases papers describing studies, especially human studies, use the term “baroreflex sensitivity” without qualification, when the authors have measured only the cardiac component of the reflex.

In animals and humans, the modified Oxford method (Smyth et al., 1969) is commonly used for assessing baroreflex function. With this method, the arterial pressure is altered independently by using vasoactive drugs while simultaneously measuring evoked reflex changes in heart rate and/or sympathetic activity. The advantage of this method is that the input-output properties of the baroreflex can be assessed over its full operating range. A limitation of the method, however, is that the reflex effects of changes in baroreceptor input on systemic arterial pressure cannot be measured. In humans, an alternative method that overcomes this limitation is to alter baroreceptor input by using a variable pressure neck collar device to change transmural pressure across the carotid sinus. This method allows measurements of the baroreflex changes in systemic arterial pressure, as well as in heart rate or sympathetic activity (Ludbrook et al., 1978, 1980; Grassi et al., 1998; Raven et al., 2006). This method also has some limitations, such as the fact that the reflex changes in arterial pressure will also alter the activity of aortic baroreceptors and thus partly buffer the carotid sinus baroreflex effects (Chapleau and Sabharwal, 2011). In addition, suction or pressure applied to the neck collar can cause discomfort and anxiety as well as evoking brief periods of apnea that may induce secondary effects (Chapleau and Sabharwal, 2011). Overall, however, both the modified Oxford method and the variable pressure neck collar method are highly effective techniques for assessing the stimulus-response relationship for the baroreceptor reflex.

Different methods are used to quantify the input-output relationship for the baroreflex. One method is to determine the regression line of best fit between the input (e.g., arterial pressure or transmural pressure across the carotid sinus) and the output (e.g., heart rate, vascular resistance, sympathetic activity, or systemic arterial pressure). The slope of this line is then taken as the gain or sensitivity of the baroreflex (e.g., Kamiya et al., 2001; Ogoh et al., 2007; Ichinose et al., 2008). A limitation of this method is that the gain of the reflex is not constant, but varies according to the operating level of arterial pressure (Raven et al., 2006). Alternatively, the input-output relationship for the baroreflex can be described by a sigmoid logistic function, as first proposed by Kent et al. (1972) (**Figure 2A**). This curve has been shown to provide a very good fit of the baroreflex input-output relationship (Kent et al., 1972; Burattini et al., 1994). It also has the advantage of providing a measure of the operating range of the reflex, i.e., the arterial pressure range over which changes in arterial pressure induce significant reflex changes in sympathetic activity or heart rate (**Figure 2A**).

**Figure 1:**
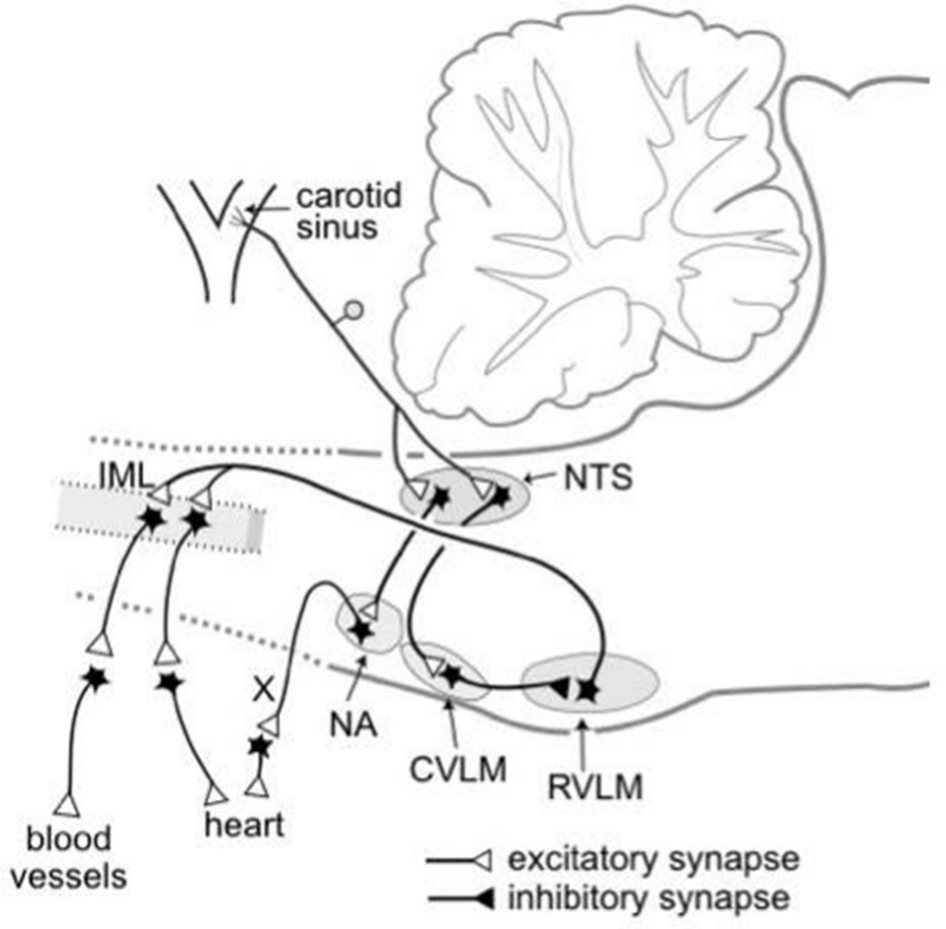
Schematic diagram showing the essential pathways that subserve the baroreflex control of the sympathetic outflow to the heart and blood vessels and the parasympathetic outflow to the heart. The baroreceptors are stretch receptors located in the walls of the carotid sinus and aortic arch (not shown). CVLM, caudal ventrolateral medulla; IML, intermediolateral cell column; NA, nucleus ambiguous; NTS, nucleus tractus solitaries; RVLM, rostral ventrolateral medulla; X, vagus nerve. Modified from Dampney (1994).

The essential central pathways that subserve the baroreflex control of sympathetic vasomotor activity are located within the medulla oblongata (Guyenet, 2006; Dampney, 2016), as shown in Figure 1. Primary glutamatergic afferents arising from baroreceptors in the carotid sinus and aortic arch terminate in the nucleus tractus solitaries (NTS), and synapse with second-order glutamatergic excitatory neurons that in turn project to and synapse with GABAergic inhibitory neurons within the caudal ventrolateral medulla (CVLM). The CVLM GABAergic neurons that are activated by baroreceptors inputs project to and inhibit sympathetic premotor neurons within the rostral ventrolateral medulla (RVLM). The barosensitive sympathetic premotor neurons in the RVLM neurons project directly to sympathetic preganglionic neurons within the intermediolateral cell column (IML) in the spinal cord, and are believed to use glutamate as their primary neurotransmitter (Guyenet, 2006). The RVLM sympathetic premotor neurons consist of various subgroups, each of which preferentially or exclusively regulates the sympathetic vasomotor outflow to different vascular beds (McAllen et al., 1995). The sympathetic vasomotor outflows to different vascular beds are influenced to different degrees by baroreceptor inputs; for example, the sympathetic outflow to renal or skeletal muscle vascular beds is strongly influenced by baroreceptor inputs, whereas the sympathetic outflow to skin blood vessels is little affected (Wallin and Charkoudian, 2007).

The nuclei within the essential central circuitry of the vasomotor baroreflex (NTS, CVLM, RVLM, and IML) also receive descending inputs from higher brain regions (Guyenet, 2006; Dampney, 2016). Such descending inputs include those that reset the vasomotor baroreflex under different behavioral conditions, as discussed in more detail in the following sections.

## Resetting of the baroreceptor-vasomotor reflex in exercise, mental stress, and sleep

### Exercise

At the onset of physical activity there is an immediate increase in arterial pressure, heart rate, and cardiac output (Rowell, 1974). Studies in conscious rats have shown that these cardiovascular changes are accompanied by increases in renal and lumbar sympathetic activity, the magnitude of which is graded according to the level of physical activity (Miki et al., 2003; Yoshimoto et al., 2010; Miki and Yoshimoto, 2013). Similarly studies in humans have shown that the sympathetic outflow to skeletal muscle vascular beds is increased during exercise (Rowell, 1997).

As mentioned in the Introduction, the parallel increases in arterial pressure and sympathetic activity led investigators to propose that the baroreflex was inhibited during exercise. As a result of careful studies carried out in both humans and animals, however, it is now known that this is not the case. In humans, Raven and co-workers have used the variable pressure neck collar to produce changes in carotid sinus transmural pressure and thus obtain logistic sigmoidal function curves describing the carotid sinus baroreflex control of systemic arterial pressure, under both resting control conditions and during different levels of exercise. These experiments showed that during dynamic exercise the baroreflex function curves were shifted upwards and to the right, thus allowing the baroreflex to continue to be operational but over a higher range of arterial pressure, corresponding to the increased arterial pressure during exercise (Papelier et al., 1994; Potts, 2006). The magnitude of this resetting varied, in accordance with the intensity of exercise. Despite this resetting, the maximum gain of the reflex was unchanged as compared to resting levels (Papelier et al., 1994; Potts, 2006). Similarly, studies in humans in which muscle sympathetic nerve activity (MSNA) was measured using microneurograpy have demonstrated that during isometric exercise the baroreflex control of MSNA is shifted to higher levels of arterial pressure, with an increase in reflex gain (Kamiya et al., 2001; Ichinose et al., 2006).

**Figure 2:**
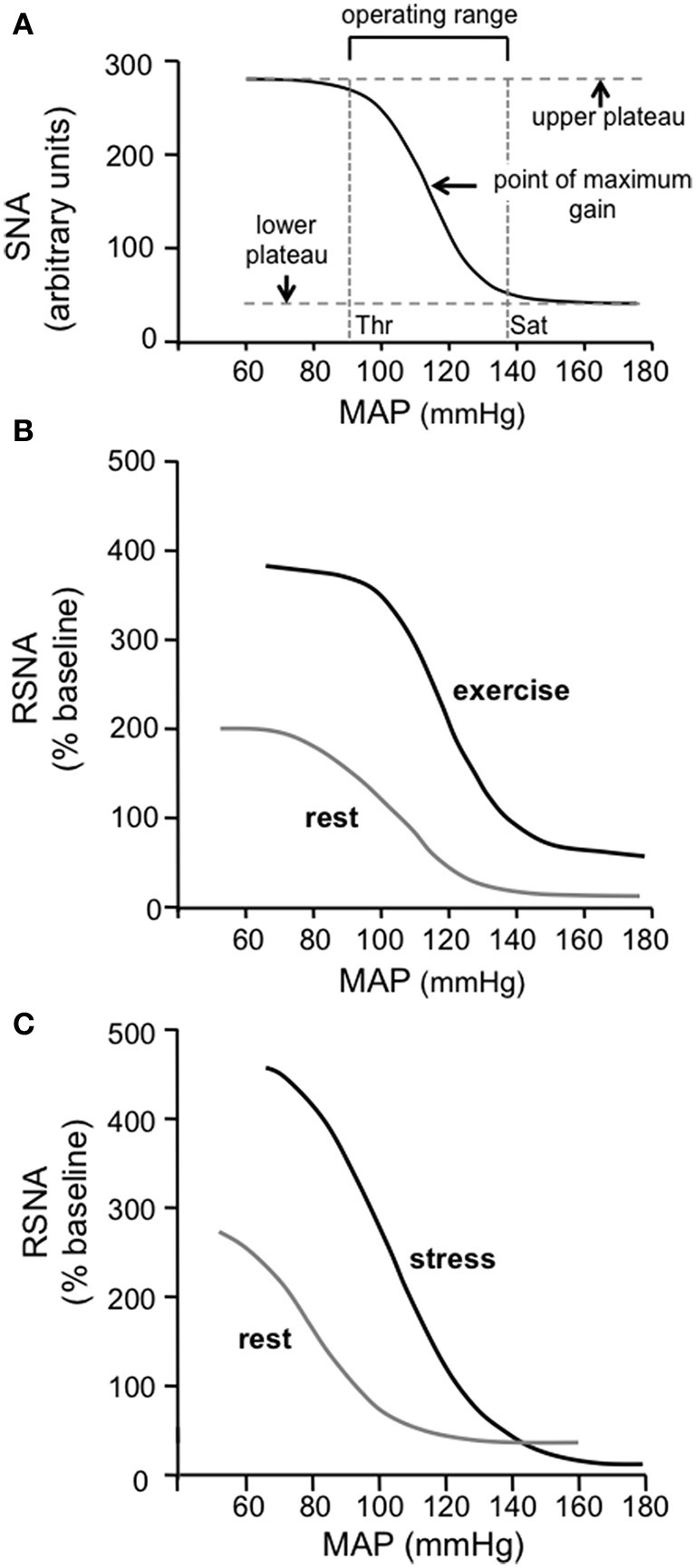
**(A)** The standard sigmoidal curve that is used to represent the input-output relationship for the baroreceptor reflex. The curve represents the following function: Y = A_1_/{1 + exp[A_2_(X - A_3_)]} + A_4_, where X is the input [mean arterial pressure (MAP) in this case] and Y is the output [sympathetic nerve activity (SNA) in this case] and A1, A2, A3, and A4 are the parameters that define the specific curve in any particular situation. The gain or sensitivity of the reflex at any value of X is represented by the slope of the curve and is maximal at the midpoint of the Y range [i.e., between the maximum (upper plateau) and minimum (lower plateau) values of Y]. The threshold (Thr) value of X is the point at which the value of Y is 5% of the Y range below the maximum value of Y, and the saturation (Sat) value of X is the point at which the value of Y is 5% of the Y range above the minimum value of Y. The operating range of X lies between the Thr and Sat values and is thus (in this example) the range of MAP over which changes in MAP evoke significant reflex changes in Y (SNA in this case). (Modified from McDowall and Dampney, 2006). **(B)** Baroreflex function curves showing the relationship between MAP and RSNA in conscious rats at rest and during exercise. Note that the maximum gain is increased and the operating range is shifted to higher values of MAP during exercise. Modified from Miki et al. (2003) with permission. **(C)** Baroreflex function curves showing the relationship between MAP and RSNA in conscious rats at rest and during psychological stress (air jet stress). Note that the maximum gain is increased and the operating range is shifted to higher values of MAP during psychological stress, similar to the changes observed in exercise. Modified from Kanbar et al. (2007) with permission.

Miki et al. (2003) determined the sigmoidal function curves describing the baroreflex control of renal sympathetic nerve activity (RSNA) in conscious rats during rest and dynamic exercise. Consistent with observations in humans, they found that the curve was shifted upwards and to the right, and that the maximum gain of the reflex was increased (Figure 2B). These studies in humans and rats thus clearly demonstrate that the baroreflex control of sympathetic vasomotor activity is not inhibited during exercise, but instead acts to regulate arterial pressure around an increased level that is physiologically advantageous during exercise.

The mechanisms that are responsible for the resetting of the baroreflex in exercise are not understood in detail. In general, however, two factors are responsible: central command and afferent inputs arising from active skeletal muscle (Rowell and O'Leary, 1990). With respect to exercise, central command refers to effects mediated by descending pathways originating from the motor cortex, while the afferents inputs from skeletal muscle arise from chemoreceptors activated by increased metabolic activity as well as mechanoreceptors that are activated by contraction of the muscles (Dampney et al., 2008). In a study in humans, Ogoh et al. (2002) used the method first described by Goodwin et al. (1972) to selectively alter central command during static exercise. With this method, the central command required to produce a given level of muscle contraction can be reduced or increased by applying vibration to the tendon of the contracting muscle or antagonist muscle, respectively. Using this method it was found that the contribution of central command to resetting of the carotid sinus baroreflex control of arterial pressure was ~50% (Ogoh et al., 2002). Similarly, various experimental approaches in both humans and animals show that inputs from muscle receptors cause resetting of the vasomotor component of the baroreceptor reflex (Raven et al., 2006). The muscle receptors that cause such resetting include both mechanoreceptors (McWilliam et al., 1991) and chemoreceptors (Papelier et al., 1997; Ichinose et al., 2008).

### Mental stress

As mentioned in the Introduction, early studies on the effects of electrical stimulation of the hypothalamic “defense area” on the baroreflex in anesthetized animals led to the belief that the baroreflex is suppressed during arousal or mental stress (Hilton, 1982). In more recent studies, however, the baroreflex control of renal sympathetic activity and heart rate were assessed in conscious rats using sigmoidal function curves, under both resting conditions and during air jet stress (Kanbar et al., 2007; Burke and Head, 2008). In contrast to the view that the baroreflex is suppressed, both these studies showed that during mental stress the baroreflex control of sympathetic vasomotor activity is reset, such that it operates over a higher range of arterial pressure with increased gain (e.g., Figure 2C).

The descending pathways that subserve baroreflex resetting during arousal or mental stress are largely unknown. It is likely that the dorsomedial hypothalamus (DMH) and adjacent perifornical area (PFA) play an important role in such resetting, because it is well-established that neurons in these regions are critical for the expression of stress-evoked cardiovascular responses (Dampney, 2015). Furthermore, disinhibition of neurons in the DMH/PFA cause resetting of the baroreflex control of sympathetic activity that closely mimics that which occurs during naturally evoked stress (McDowall et al., 2006).

### Sleep

During both the rapid-eye movement (REM) and non-REM phases of sleep in rats, the baroreflex-renal sympathetic function curve is shifted downwards and to the left, so that it operates over lower levels of arterial pressure and RSNA (Nagura et al., 2004). In humans the baroreflex control of MSNA is altered in a similar fashion during sleep (Sayk et al., 2007). This shift is due to central resetting of the baroreflex, rather than to adaptation of the baroreceptors themselves to the lower arterial pressure that occurs during sleep (Sayk et al., 2007). As with exercise and mental stress, the descending pathways that subserve baroreflex resetting during sleep are largely unknown (Silvani and Dampney, 2013).

In summary, the resetting of the baroreflex control of sympathetic vasomotor activity is not fixed but changes according to the behavioral state. The effect of this is that the arterial pressure at all times is regulated around a level that is appropriate for the particular behavioral state. For example, during exercise or mental stress the increased level of arterial pressure allows for increased blood flow to the skeletal muscle and the heart in response to an actual or potential increased metabolic demand. In contrast, the decreased arterial pressure during sleep has the effect of reducing cardiac work while maintaining an adequate perfusion of vital organs.

In many behaviors there is a gradual change from the resting state to the new behavioral state. For example, during sustained exercise at a constant level there is a continual increase in muscle fatigue, which would be expected to result in a gradually increasing input from both peripheral receptors and central command. As pointed out by Zamir et al. (2017) in another article in this Research Topic, it is likely that under those conditions the baroreflex would be reset in a gradual fashion. Thus, while experimental determinations of the baroreflex function curves are typically made at particular time points (e.g., at rest and at some time during the exercise), it should be emphasized that baroreflex resetting is a continuous process rather than a sudden change from one state to another.

## Mechanisms of resetting within the vasomotor baroreflex circuitry

As mentioned above and illustrated in Figure 1B, the key central nuclei of the central circuitry mediating the baroreflex control of sympathetic activity are the NTS, CVLM, RVLM, and the IML in the thoracolumbar spinal cord that contains sympathetic preganglionic neurons (SPN). Anatomical and electrophysiological evidence indicates that the connections subserving the baroreflex within each of these nuclei are monosynaptic (Aicher et al., 1995, 2000; Bailey, 2006). Thus, resetting of the baroreflex can only occur via modulation of synaptic transmission within one or more of these key nuclei.

There are four ways in which the baroreflex control of sympathetic vasomotor activity can be reset, as described by the baroreflex function curve: (1) the curve can be shifted laterally to the left or right, corresponding to a shift in the operating range of arterial pressure (to higher and lower values, respectively) in which reflex changes in sympathetic activity can be evoked; (2) the upper plateau of the curve can be shifted vertically up or down, which corresponds to an increase or decrease in sympathetic activity when the baroreceptor afferent input is below the threshold level; (3) the maximum slope of the curve can be increased or decreased, corresponding to an increase or decrease, respectively, in the gain or sensitivity of the reflex; (4) the lower plateau of the curve can be shifted vertically up or down, which corresponds to an increase or decrease in sympathetic activity when the baroreceptor afferent input is above the saturation level. For example, during exercise in conscious rats Miki et al. (2003) found that the baroreflex sympathetic function curve was reset in such a way that all four of these parameters were increased (Figure 2B).

**Figure 3:**
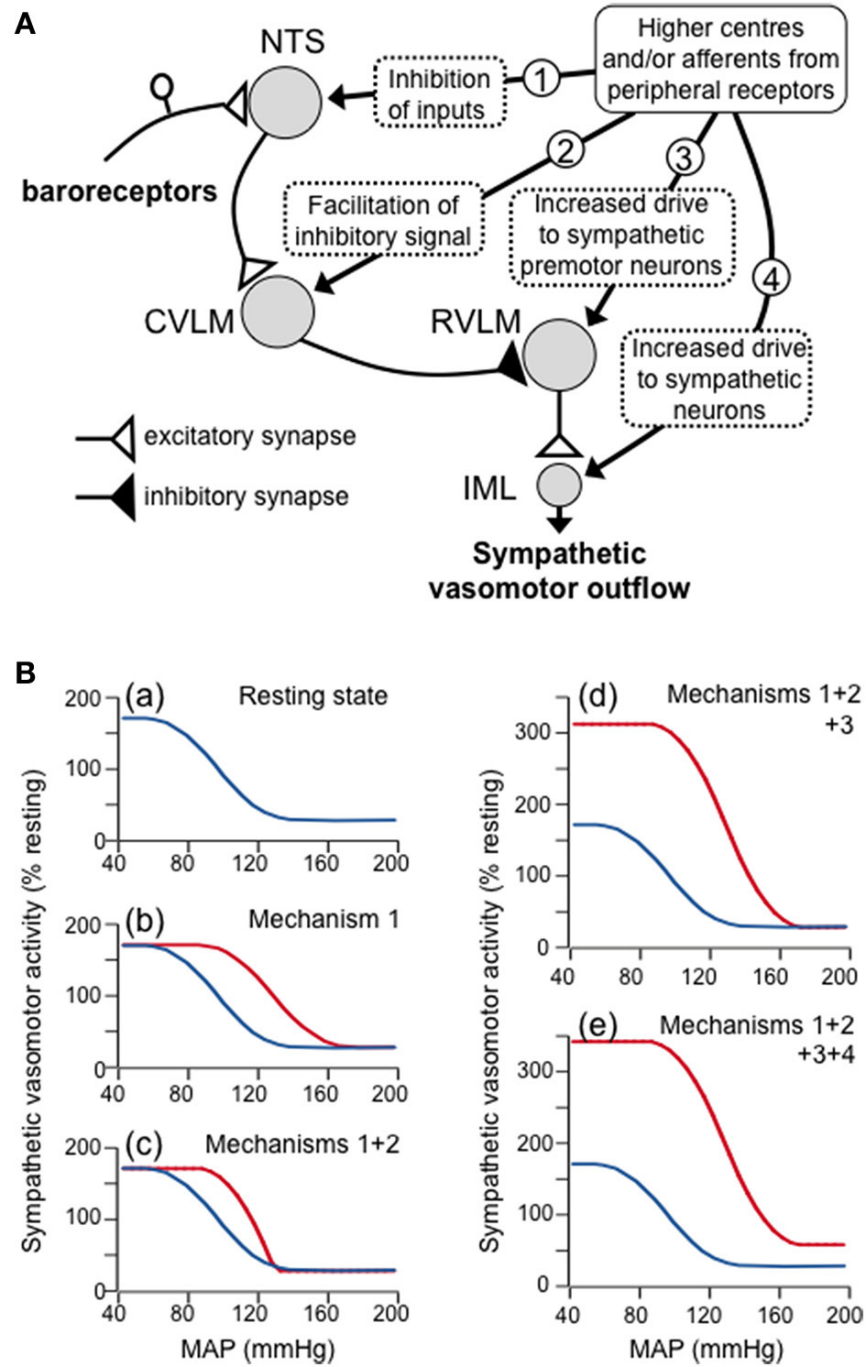
**(A)** Schematic diagram showing a proposed model of the four mechanisms by which higher centers in the brain and/or afferents from peripheral receptors can produce resetting of the baroreflex control of the sympathetic vasomotor outflow. These four mechanisms are indicated by the circled numbers in the diagram, and are: (1) inhibitory inputs to second-order barosensitive neurons in the NTS - these have the effect of shifting the baroreflex function curve to the right; (2) inputs to GABAergic neurons in the CVLM that facilitate the excitatory inputs to these neurons from second-order barosensitive neurons - these have the effect of increasing the slope of the baroreflex function curve; (3) excitatory inputs that increases the activity of sympathetic premotor neurons in the RVLM - these have the effect of raising the upper plateau of the baroreflex function curve; (4) excitatory inputs to sympathetic vasomotor preganglionic neurons in the spinal cord IML that are independent of the baroreceptor reflex - these have the effect of raising both the upper and lower plateaus of the baroreflex function curve. **(B)** Baroreflex function curves as derived from the model, showing (a) the baroreflex curve under resting conditions, and the effects of increasing (b) mechanism 1, (c) mechanisms 1 and 2, (d) mechanisms 1, 2 and 3, and (e) mechanisms 1, 2, 3, and 4. Note that the combined effect of all these mechanisms simulates the shift in the baroreflex function curve that occurs during exercise.

Figure 3A is a conceptual model that is capable of reproducing any of the four types of resetting described above. In this model, it is proposed that there are inputs to barosensitive neurons within all four key nuclei (NTS, CVLM, RVLM, and IML) that arise either from higher centers in the brain or from peripheral receptors (e.g., muscle receptors) that may be activated in different behavioral states. In particular, the second-order neurons within the NTS in this model receive convergent excitatory inputs from primary baroreceptor afferent fibers, and inhibitory GABAergic inputs from other sources, consistent with the known properties of these neurons (Potts, 2006; McDougall and Andresen, 2012). The activity of these second-order neurons, therefore, depends upon the net effect of the excitatory and inhibitory inputs to the neurons. Second, the model also includes an input to CVLM neurons from higher centers and/or peripheral receptors, which are postulated to facilitate inhibitory inputs from the second-order NTS barosensitive neurons. Consistent with this model, the activity of CVLM barosensitive neurons can be modulated by inputs from sources other than the NTS, such as from central inspiratory neurons (Mandel and Schreihofer, 2006), and this modulation has the effect of facilitating CVLM inhibitory inputs to RVLM neurons (Miyawaki et al., 1995). Third, it is well-established that there are excitatory inputs to RVLM sympathetic premotor neurons from many sources (Guyenet, 2006; Dampney, 2016) and these are also included in the model. Finally, the model also includes descending excitatory inputs to SPN in the spinal cord IML that do not synapse in the RVLM, and which are independent of the baroreflex pathway.

The conceptual model in Figure 3A can also be presented as a mathematical model, so that the effects on the baroreflex of changing the magnitude of any of the four modulatory outputs (labeled 1–4 in Figure 3A) from higher centers and/or muscle afferents can be determined. For example, Figure 3B shows how this model can mimic the baroreflex resetting that occurs during exercise (e.g., Figure 2B). At rest, it is assumed that none of the inputs from higher centers and/or muscle afferents to the NTS, CVLM, RVLM, and IML are active, with the exception, of a tonic excitatory input to RVLM neurons (Figure 3Ba). Under those circumstances, second-order neurons in the NTS respond only to primary baroreceptor afferent inputs. During exercise, however, it is assumed that GABAergic inputs (arising from muscle receptors and/or higher centers; Potts, 2006) become active, so that a higher level of primary baroreceptor activity (and therefore arterial pressure) is required before the second-order neurons reach threshold. Similarly, the arterial pressure at which second-order neurons reach their saturation level will also be increased. When such an inhibitory input is included in the model, the baroreflex function curve is shifted to the right (Figure 3Bb).

Second, it is assumed that during exercise the excitatory input from second-order NTS neurons to CVLM neurons is facilitated (i.e., there is a greater change in CVLM neuron activity for the same change in input). When this is included in the model, the slope of the baroreflex function curve is increased (i.e., the maximal gain is increased) (Figure 3Bc). Third, it is also assumed that during exercise the excitatory input to RVLM sympathetic premotor neurons, arising from higher centers and/or skeletal muscle afferents (Potts, 2006) is increased. This has the effect of increasing the upper plateau of the baroreflex function curve (Figure 3Bd). Finally, an increase in descending drive to sympathetic preganglionic neurons, independently of the baroreflex pathway, will increase both the lower and upper plateau levels of the baroreflex function curve (Figure 3Be). Taken together, all of these changes result in a new baroreflex function curve that mimics the effect of exercise.

Other models may also be capable of mimicking baroreflex resetting in exercise or during other behaviors. Nevertheless, the model presented in Figure 3 is perhaps the simplest one that is consistent with the known properties of the central baroreflex circuitry and its modulatory inputs. Future studies are required, however, to define precisely the central mechanisms that subserve baroreflex modulation.

## Summary and conclusions

The baroreceptor reflex is the single most important reflex regulating arterial pressure, at least in the short term. The reflex regulates arterial pressure primarily via changes in sympathetic vasomotor activity. The functional characteristics of the baroreflex control of sympathetic vasomotor activity, however, are not constant, but are constantly changing according to the behavioral state of the animal. The effect of this is that baroreflex acts to regulate the arterial pressure around a level that is appropriate for the particular behavioral state. There are two general mechanisms that are responsible for baroreflex resetting—control command and feedback from other peripheral receptors (e.g., muscle receptors signaling changes in metabolic activity). The central pathways that subserve such resetting are poorly understood, although some information is available with respect to baroreflex resetting in exercise. In particular, there is good evidence that the NTS is a site at which modification of transmission of baroreceptor signals occurs in exercise and probably also in other behaviors. It is difficult to explain all the different ways in which baroreflex resetting may occur, however, solely as a consequence of modulatory effects at the level of the NTS. In this review I have therefore proposed a model in which modulatory changes can occur at all of the key regions that constitute the core circuitry of the baroreceptor-vasomotor reflex: the NTS, CVLM, RVLM, and IML. This model can simulate all the components of baroreflex resetting. Future studies are required to determine whether this proposed model is an accurate representation of the central mechanisms responsible for baroreflex resetting. Further studies are also required to identify the nuclei and neural pathways at all levels of the brain that regulate the baroreflex.

## Author contributions

The author confirms being the sole contributor of this work and approved it for publication.

### Conflict of interest statement

The author declares that the research was conducted in the absence of any commercial or financial relationships that could be construed as a potential conflict of interest.
